# Exploring perceived relationships between weather, climate and mental health: biometeorological perspectives of healthcare practitioners

**DOI:** 10.1007/s00484-024-02791-6

**Published:** 2024-10-09

**Authors:** Mukhtaar Waja, Jennifer M. Fitchett

**Affiliations:** School of Geography, Archaeology and Environmental Studies, University of the Witwatersrand, Johannesburg, South Africa

**Keywords:** Mental health, Climate, Climate change, Temperature, Extreme weather events, Seasonal affective disorder

## Abstract

Over the last decade, there has been an increase in research examining the influence of weather and climate in mental health caseloads. Variations in temperature, sunshine hours, cloud cover, precipitation and extreme weather events have been statistically linked to diagnoses and increases in hospital admissions for several mental health conditions. This study aimed to explore whether mental health practitioners perceive there to be a link between mental health and daily, seasonal, or inter-annual shifts in various climate variables in South Africa, and the timing and causal mechanisms thereof. Semi-structured interviews were conducted with 50 practicing healthcare practitioners, and the data was analysed using thematic analysis. The findings of this research show that all 50 participants were aware of the link between weather, climate and mental health, primarily through their awareness of seasonal affective disorder. Of the 50 participants, 38 participants could explain the aetiology of seasonal affective disorder. Participants perceived sunlight and temperature to exert an influence on mental health. All 50 participants perceived exposure to sunlight to exert a positive influence on several mental health conditions. Of the 50 participants, 36 participants perceived increases in temperature to exert an adverse effect on mental health symptomology. A minority of 11 participants perceived precipitation to influence mental health conditions such as seasonal affective disorder, bipolar disorder, and substance abuse disorder. Participants’ perceptions of the influence of precipitation on mental health provided a unique potential explanation of this relationship, which, at the time of writing, has not been discussed in formal research.

## Introduction

Mental health challenges are of great relevance in the South African context because of its contribution to the burden of disease (Craig et al. 2022). Research suggests that the prevalence of mental health conditions throughout South Africa is greater than other low- and middle-income countries (Flisher et al. 2012; Craig et al. 2022). The most prevalent mental health conditions throughout South Africa include generalised anxiety disorder (GAD), substance abuse disorder, major depressive disorder, and post-traumatic stress disorder (PTSD; Nguse and Wassenaar 2021). The combination of crime rates, gender-based violence, unemployment, adolescent pregnancy, and a high prevalence of communicable diseases pose major threats to the mental health of South African citizens (Byansi et al. 2023).

Over the recent decade, there has been an increase of weather and climate in the incidence of mental health related diagnoses (Maes et al. 1993; Bulbena et al. 2005; Jahan et al. 2020; Bundo et al. 2021; Liu et al. 2021). The most widely understood and well-documented link between weather and climate and a mental health related diagnosis is Seasonal Affective Disorder (SAD; Magnusson and Boivin 2003; Melrose 2015; Parker et al. 2017). Studies have exhibited statistically significant relationships between variations in temperature, sunlight exposure, and rainfall and mental health conditions such as schizophrenia, bipolar disorder, major depressive disorder, and GAD (McWilliams et al. 2014; Bundo et al. 2021; Liu et al. 2021). However, many of these studies have been criticized for the datasets used, statistical approaches to analysis, and the representativity of results (Brandl et al. 2018; Bundo et al. 2021; Liu et al. 2021). Furthermore, many of these studies remain within the confines of scientific journals, often in the domains of applied climate sciences, and thus specialist medical clinicians who treat mental illness may not be aware of or up to date on this research.

Interventions and policies that address mental health care need to address the multidimensionality of mental health challenges, and thus its various risk factors must be considered – including genetic predisposition, issues surrounding socioeconomic status, and possible climatic risk factors (Jahan et al. 2020; Clayton 2021). Research is lacking on whether mental health practitioners in South Africa perceive there to be a link between various weather and climaticvariables and mental health conditions, and whether they have any documented patterns to support this in their medical practise. This research fills the gap in the literature by studying this topic, while embracing the multi-cultured and unique perspectives of mental health practitioners in South Africa.

## Materials and methods

### Study region

**Fig. 1:**
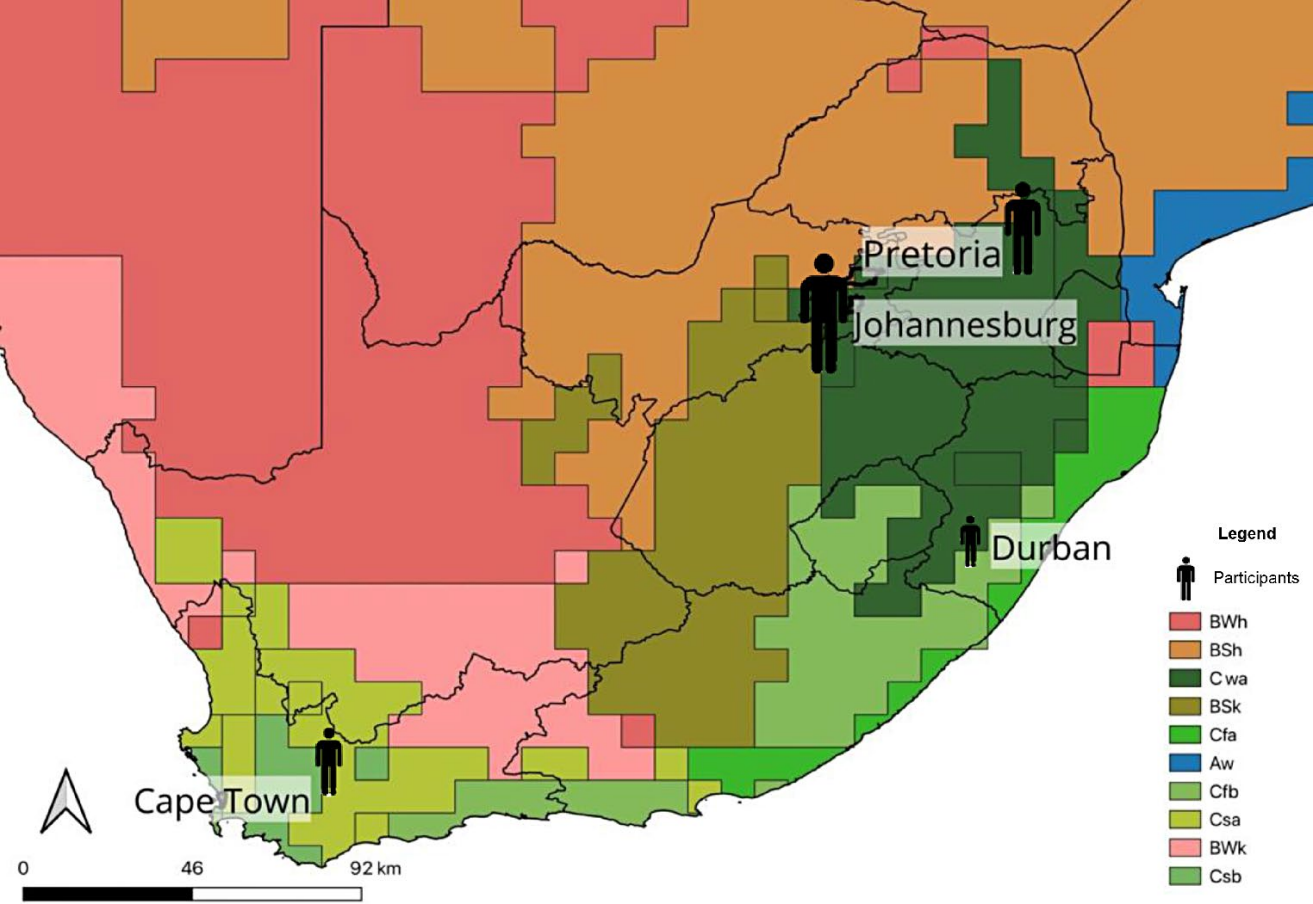
Koppen-Geiger map of South Africa depicting the climate zones in relation to participants’ city of practice. The size of the participant icon reflects the amount of participants who practiced in each city

South Africa is located within the southern Hemisphere, at the southernmost tip of Africa, and is characterised by a diverse climate (Karmalkar et al. 2012). The southwestern region of South Africa generally experiences winter rainfall during the winter months from May to August, while the northern interior experiences predominantly summer rainfall from November to March (Nicholson 2000; Roffe et al. 2021). Temperature differences along the east and west coasts of South Africa are also influenced by the impact of the respective ocean currents along each coast: air temperatures along the east coast tend to be warmer than those of the west coast, due to the warming effect of the Agulhas Current and the cooling effect of the of the Benguela Current respectively (Tim et al. 2022). A plateau elevates a large section of the interior of South Africa, and as a result, cooler temperatures are experienced on the plateau (Fig. 1; Karmalkar et al. 2012), with a large temperature range where summer temperatures can rise above 32ºC, and can drop below freezing point at certain high elevations (Fitchett et al. 2016).

There are numerous mental health risks in South Africa that may explain the high prevalence of mental health challenges in the local context, and which may be exacerbated by climate change (Atwoli et al. 2022; Craig et al. 2022). Within South Africa there are high rates of poverty, unemployment, crime, violence, adolescent pregnancy, and communicable diseases that have a high comorbidity with mental health conditions (Posel et al. 2021; Craig et al. 2022). Individuals exposed to violence, abuse or neglect during their adolescence are more likely to experience mental health challenges than the general population, being vulnerable to PTSD, depression, and anxiety (Craig et al. 2022). Although mental healthcare was made a priority in the Sustainable Development Goals, Johannesburg’s healthcare infrastructure and resource accessibility are severely limited (Docrat et al. 2019; Robertson et al. 2024). The gap between policy and practice, with regards to South Africa’s treatment of mental health, is still large, with many public mental health facilities underfunded and understaffed (Pillay 2019).

### Data collection

This study was conducted through a series of semi-structured interviews. Sem-structured interviews were conducted with 50 medical practitioners working in the field of mental health in South Africa, comprising 10 participants from each of the following categories: general practitioners, emergency physicians, psychologists, psychiatrists, and social workers. Thus, participants were selected based on their profession falling within one of these five selected medical fields. Knowledge of the relationship between weather, climate and mental health was not a part of the criteria for participation, and the participants did not have any known prior knowledge of this relationship. A combination of non-probability purposive, quota and snowball sampling with clear criteria for participation was adopted. Initial participants were identified through convenience sampling – utilising personal networks and connections to participants who met the participation criteria – and by contacting healthcare facilities. Following this, participants were asked to recommend other possible participants, who they believed met the criteria for participation, that of being a practicing medical professional within one of the five specified medical fields (Etikan and Bala 2017). Before data collection commenced, ethical clearance was granted by the WITS Human Medical Ethics Committee (reference number: M220601). This study did not utilise data from medical records and focused solely on data collected from the semi-structured interviews. All participants’ responses were anonymised, and the only demographic information which was captured were participants’ profession, years of experience, and city of practice.

The questions examined key themes relating to this research namely participants’ awareness of the relationship between weather, climate and mental health and the existence of literature examining this relationship, observation of seasonal peaks in mental health conditions throughout the year and importantly their perception of the climate sensitivity of mental health conditions, which weather and climatic conditions impact mental health conditions and to explain what they perceived the aetiology behind this potential impact to be. In addition, the consideration of the climate context (such as whether the patient is in a summer or winter rainfall zone), and recent weather conditions (such as the amount of cloud cover during the week prior to treatment) when examining mental health conditions was examined. Participants’ understanding of the phrase “climate context” and “weather conditions” was informed by the climatic and weather variables outlined in the interview questions, which included seasonal variations and daily or weekly fluctuations in day-time temperature, night-time temperature, rainfall, sunshine hours, cloud cover, wind, and relative humidity. Participants were given the opportunity to discuss other weather and climatic variables, which they perceived to impact mental health, but none were suggested.

The data in this study were analysed by means of thematic analysis. Both inductive and deductive thematic analysis were utilised for this study, drawing on both published research on the relationship between weather, climate and mental health, and participants’ responses to inform the themes and codes identified in the data (Adeoye-Olatunde and Olenik 2021). Audio recordings from interviews were manually transcribed, and through the process of thematic coding, clearly defined themes emerged (Braun and Clarke 2006).

## Results

### Participant demographics

The demographics of the participant group (Table 1) are worth reporting yet demonstrated no clear explanatory role in the differences in responses obtained. The range in participants’ years of professional experience is large, even among the different categories of healthcare professionals (Table 1). The participant with the least years of experience had four years of experience, while the participant with most years of experience had 38 years of experience. The majority of participants in this study possessed either Masters degrees or PHDs, with some participants having additional diplomas in their field of study (Table 1). Degrees held by the social workers were more varied.

**Table 1:** The profession, years of professional experience and qualification of participants

Participant	Profession	Years of experience	Qualifications
1 A	General Practitioner	30	MMed in Family Medicine
1B	General Practitioner	6	MBChB
1 C	General Practitioner	14	MBChB
1D	General Practitioner	23	MBChB
1E	General Practitioner	14	MBChB
1 F	General Practitioner	9	MBChB and MMed in Public Health Medicine
1G	General Practitioner	20	MBChB
1 H	General Practitioner	27	MBChB and a Diploma in Family Medicine
1I	General Practitioner	25	MBChB and a Masters Degree in Sports Medicine
1 J	General Practitioner	10	MBChB
2 A	Emergency physician	8	MMed in Emergency Medicine
2B	Emergency physician	10	MMed in Emergency Medicine and Diploma in Emergency Care
2 C	Emergency physician	5	MMed in Emergency Medicine
2D	Emergency physician	23	MMed in Emergency Medicine and Fellowship of Emergency Medicine
2E	Emergency physician	12	MMed in Emergency Medicine
2 F	Emergency physician	10	MMed in Emergency Medicine
2G	Emergency physician	4	MMed in Emergency Medicine
2 H	Emergency physician	7	MMed in Emergency Medicine
2I	Emergency physician	20	MMed in Emergency Medicine
2 J	Emergency physician	10	MMed in Emergency Medicine
3 A	Psychologist	18	PhD Clinical Psychology
3B	Psychologist	25	PhD Psychology
3 C	Psychologist	25	PhD Psychology
3D	Psychologist	30	PhD Clinical Psychology
3E	Psychologist	29	PhD Clinical Psychology
3 F	Psychologist	10	PhD Psychology
3G	Psychologist	13	PhD Psychology
3 H	Psychologist	10	PhD Psychology
3I	Psychologist	38	PhD Psychology
3 J	Psychologist	10	PhD Psychology
4 A	Psychiatrist	37	PhD Psychiatry
4B	Psychiatrist	7	MMed Psychiatry
4 C	Psychiatrist	15	MMed Psychiatry
4D	Psychiatrist	13	MMed Psychiatry
4E	Psychiatrist	35	PhD Psychiatry
4 F	Psychiatrist	5	MMed Psychiatry
4G	Psychiatrist	9	MMed Psychiatry
4 H	Psychiatrist	25	MMed Psychiatry and Diploma in Mental Health Studies
4I	Psychiatrist	10	MMed Psychiatry
4 J	Psychiatrist	7	MMed Psychiatry
5 A	Social Worker	~ 35	MA Psychology
5B	Social Worker	30	BA Honours in Psychology
5 C	Social Worker	27	Bachelor of Education
5D	Social Worker	35	BA Honours in Psychology
5E	Social Worker	25	Bachelor of Social Work
5 F	Social Worker	32	Bachelor of Social Work
5G	Social Worker	17	BA Honours in Psychology
5 H	Social Worker	20	BEd Honours
5I	Social Worker	30	Master of Social Work
5 J	Social Worker	5	BA Honours in Psychology and Bachelor of Social Work

### Awareness of the relationship between weather, climate and mental health

Participants were asked if they were aware of the potential existence of a relationship between weather, climate and mental health, and if they were familiar with the literature on the subject. All 50 participants interviewed were aware of the existence of SAD, and therefore were indeed aware of a link between weather, climate and mental health. Participants’ descriptions were consistent with the formal diagnosis of SAD, referencing the effects of reduced sunlight on serotonin, melatonin, and circadian rhythm. Of the 50 participants, 38 displayed an understanding of the aetiology of SAD, either drawing on their knowledge of the condition, or through applying their knowledge of medical science to discern the potential aetiology (Fig. 1). Aside from SAD, the majority of participants were not aware of the potential influence that average weather and climate could exert on other mental health conditions, such as bipolar disorder, schizophrenia, and GAD (Fig. 1). However, 36 participants were able to apply their medical knowledge to discern the manner in which temperature may affect such mental health conditions. Only two participants were familiar with the published research that had been conducted on the relationship between weather, climate and mental health, and a separate four participants had read news articles covering and summarising research on this relationship. The remaining 44 participants were unaware of any literature on this topic.

**Fig. 2:**
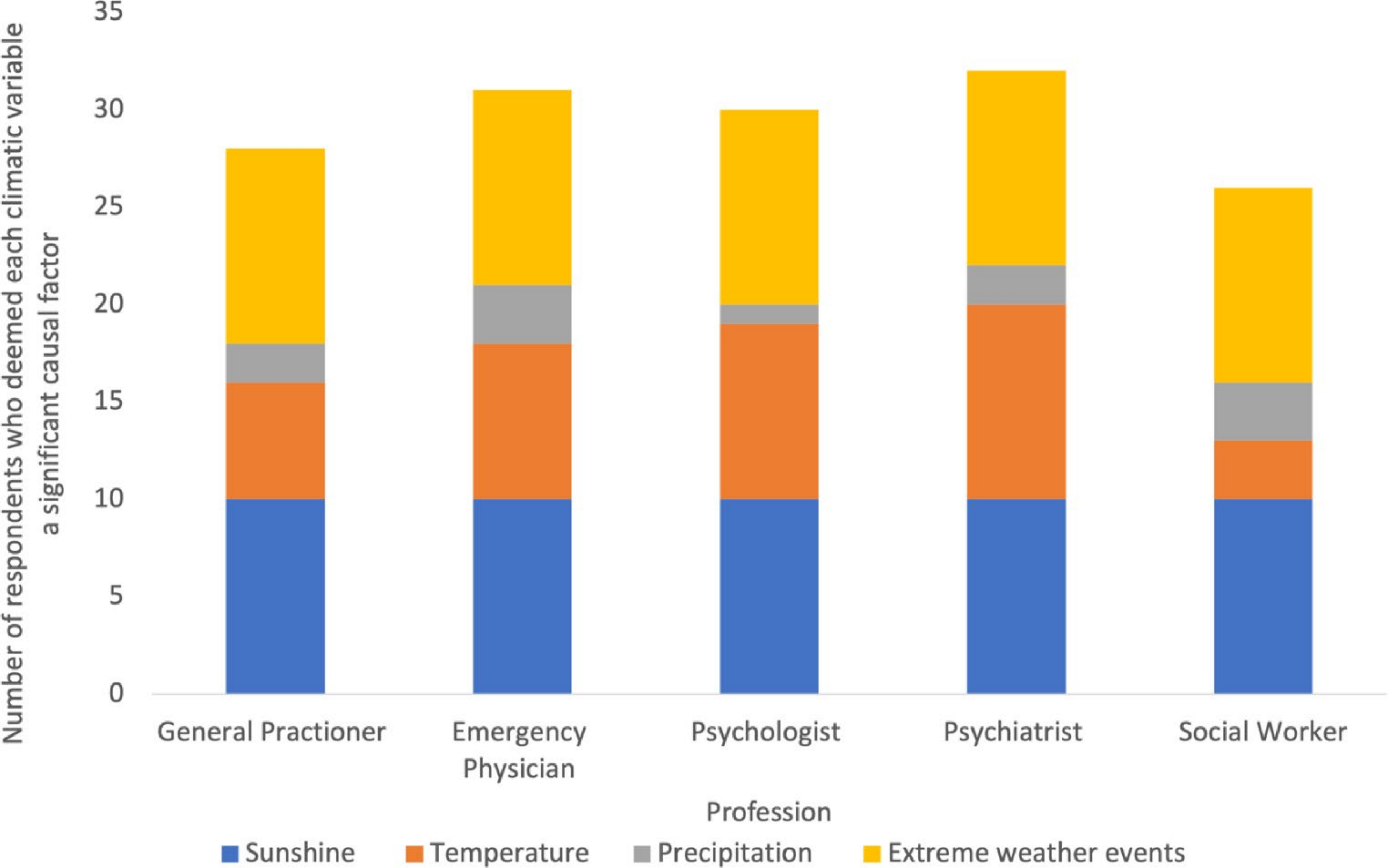
Participants’ perception of the impact of sunshine, temperature, precipitation, and extreme weather events on mental health

All 50 participants were aware of the importance of sunlight, and its potential role in treating depression through light therapy. All 50 participants also understood the impact of extreme weather events on mental health, with all participants acknowledging the multiple pathways through which these events could impact mental health and wellbeing. Only 11 participants considered the potential impact of precipitation on mental health, with ten participants focusing on social pathways as opposed to biological pathways (Fig. 2). Although all participants expressed a belief that weather and climate do influence incidence of mental health, participants stressed the importance of acknowledging the complexity and multidimensionality of mental health – emphasising the importance of considering social, physical, environmental and psychological influences. Thus, participants’ perceptions of the significance of the influence of weather and climate on mental health is contested.

### Mental health conditions perceived to be impacted by climate and weather

Participants were asked which mental health conditions displayed pronounced seasonality, or sensitivity to specific climatic variables. The responses varied based on their profession and experience with the specific mental health conditions. Emergency physicians appeared more familiar with substance abuse disorder, and the effect of weather on crime and agitation, while psychologists and psychiatrists discussed the potential impacts of weather and climate on SAD, bipolar disorder, schizophrenia, and PTSD. Both general practitioners and social workers did not have any comments regarding bipolar disorder or schizophrenia but did provide insights into the influence of weather and climate on SAD, substance abuse disorder, and PTSD.

Decreased exposure to sunlight during winter was perceived to be the main factor driving incidences of SAD, leading to serotonin dysregulation, vitamin D deficiency, and circadian dysfunction, thus explaining the biological pathways by which sunlight exposure may impact depression. The social pathways by which weather and climate may influence SAD was discussed by fewer participants, and those who did discuss it suggested that cold, rainy weather might lead to social isolation, as an individual would be less likely to leave their home, leading to an increase in depressive symptoms. Four participants stated that while they do believe that sunlight is useful in treating depression, major depressive disorder and SAD stems more from social factors, and it is these factors which should be the focus during treatment, to avoid mistreating patients.

**Fig. 3:**
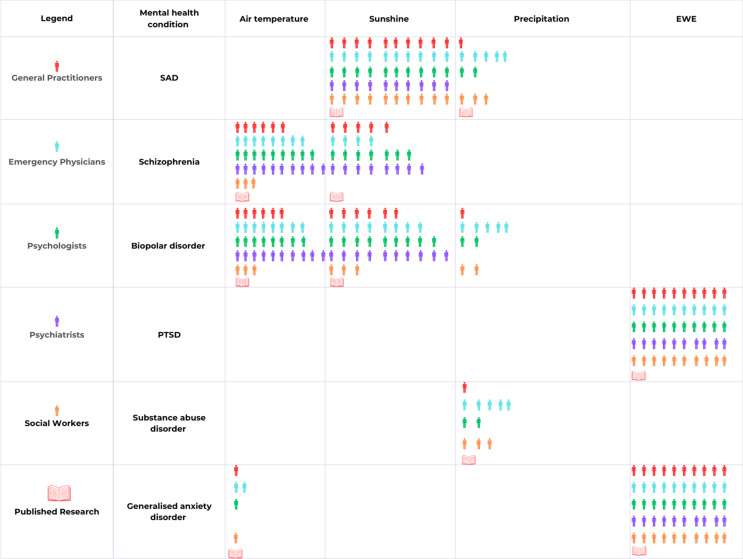
Infographic depicting the number of participants, organised by medical profession, who associated specific weather variables with mental conditions, and whether the majority of published research supports this association

Of the 50 participants interviewed, none had noticed a distinct seasonality in incidences of schizophrenia. Of the 50 participants, 36 participants were able to apply their medical knowledge of schizophrenia to discern the manner in which temperature may affect the condition (Fig. 3). Participants argued that night-time temperature may exert an important influence on schizophrenia, as well as on bipolar disorder and GAD. Individuals diagnosed with schizophrenia are more sensitive to heat stress, potentially due to the side effects of antipsychotic medication. There is a conflicting perception about the impact of sunlight on schizophrenia, with some participants arguing that an increase in serotonin production is harmful, whilst other participants suggest that exposure to sunlight can be beneficial to schizophrenic patients who struggle with a vitamin D deficiency.

None of the 50 participants noticed a seasonality in incidences of bipolar disorder in their medical practise. Utilising prior knowledge of the condition and its aetiology, 36 out of the 50 participants were able to suggest ways weather and climate may impact bipolar disorder. The impact of sunlight and temperature on bipolar disorder was discussed by 36 participants, all of whom mentioned the impacts of sunlight and temperature on circadian dysfunction, and serotonin and melatonin levels.

All 50 participants understood the multiple pathways by which extreme weather events could impact rates of PTSD, and associated extreme weather events with increased rates of PTSD, anxiety and depression. It was noted that financially poor populations and children were most vulnerable to the psychological impacts of extreme weather events, with PTSD and GAD being commonly associated with extreme weather events.

### Familiarity with research on weather, climate and mental health

Four of the 50 participants interviewed were aware of news articles summarising research on the relationship between violent crimes and increases in temperature and agreed with the findings. Participants highlighted that heat stress results in conflict and violence, indicating how weather and climate can impact an individual’s social environment.

None of the participants interviewed were familiar with the research surrounding the relationship between weather, climate and substance abuse disorder. Out of the sample participants, 18 participants considered substance abuse disorder as sensitive to the influence of weather and climate – incidences of substance abuse disorder would increase when experiencing cold weather and heavy precipitation. Due to socioeconomic disadvantages in poor communities, substance abuse can be viewed as a coping mechanism.

All 50 participants agreed that it is difficult to conclusively deny that confounding factors – such as neurological predispositions or socioeconomic circumstances – may be responsible for the results attributed to weather and climate. It was suggested that confounding factors exert a more significant influence on mental health than weather and climatic variables, and that many of these risk factors likely act independently of weather and climatic variables. This perception stems from the need to avoid mistreating patients by focusing on less significant risk factors. Of the 50 participants, 28 perceived the influence of weather and climate on mental health to be important, acknowledging that weather and climatic risk factors likely work in tandem with other risk factors.

Participants responses highlights the way in which participants are able to apply their knowledge of medical science and mental health to draw conclusions regarding the relationship between weather, climate and mental health. It also highlights the fact that these conclusions are constrained by a lack of familiarity with research on this relationship, and limited knowledge of the aetiology of complex mental health conditions.

## Discussion

### Influence of weather and climate on mental health

All 50 participants interviewed for this study perceived there to be a link between weather, climate and metal health, and participants’ perceptions of the potential aetiology behind this relationship were largely in line with the published research (Mullins and White 2019; Schneider et al. 2020; Liu et al. 2021). Similar to the patterns in the literature (Maes et al. 1993; Bulbena et al. 2005; Jahan et al. 2020; Liu et al. 2021), participants’ perceptions regarding the significance of this relationship were divergent. The perspective of a large minority of participants that weather and climate may impact mental health conditions, but that its influence is relatively insignificant compared to confounding factors, reflects a conceptualisation of mental health conditions as products of socioeconomic stresses and biological and familial predispositions (Allen et al. 2014; Compton and Shim 2015; Muneer 2016). Medical research on the aetiology and treatment of mental health conditions supports this conception (Muntaner et al. 2015; Muneer 2016), and the participants in this current study appeared to be more familiar with this conceptualisation of mental health conditions and the treatment thereof. While the importance of addressing socioeconomic and biological determinants of mental health was acknowledged by all participants, the majority of participants perceived weather and climate to exert a considerable influence on mental health, while acknowledging that it works in tandem with socioeconomic and physio-psychological factors. Participants argued that the risks and benefits that specific meteorological conditions pose towards mental health was of relative importance and should be considered more by healthcare professionals – a view which aligns with the majority of research on this topic (Basu et al. 2018; Lee et al. 2018; Mullins and White 2019; Zhang et al. 2020).

The findings of this study suggest that professionals perceive temperature and mental health are linked, which supports research findings suggesting increased air temperatures may exacerbate the symptoms of schizophrenia, bipolar disorder, GAD, panic disorders, and neurotic conditions (Yi et al. 2019; Niu et al. 2020). Based on their medical knowledge of the impact of temperature on the human body, 36 participants perceived air temperature to be an important weather variable with the potential to adversely impact almost all mental health conditions, although participants discussed the impact of air temperature on schizophrenia and bipolar disorder more than other conditions. The findings of this study suggest that high nocturnal temperatures may adversely impact the body’s thermoregulation, causing circadian dysfunction, leading to a disruption in the body’s production of serotonin and dopamine, thus exacerbating symptoms of various mental health conditions (Wang et al. 2018; Zheng et al. 2019; Jahan et al. 2020, 2021). Based on participants’ responses, and the findings of published research, it can be argued that nocturnal temperature may be more harmful than daytime temperature, due to the considerable effect of air temperature on circadian rhythm, and the role of circadian dysfunction in many mental health conditions (Sung et al. 2011; Lõhmus 2018; Zheng et al. 2019).

Of the 50 participants interviewed for this study, five participants were familiar with the research associating increased air temperatures with increased rates of violent crimes (Schutte and Breetzke 2018), and the participants’ responses supported its findings that the adverse effect of heat on sleep quality and quantity may lead to impaired cognitive function and irritability, inspiring hostile and combative behaviour (Chersich et al. 2019; Mullins and White 2019; Potgieter et al. 2022). Participants argued that warmer air temperatures affect individual activities in ways that precipitate social interactions, which, coupled with the increased propensity for violent or aggressive behaviour, increases the chances of a conflict occurring (Schutte and Breetzke 2018; Chersich et al. 2019). Participants thus suggested that increased air temperature may impact anxiety and panic disorders due to the influence of high air temperatures on social behaviour, which is not only supported by previous research (Mullins and White 2019; Potgieter et al. 2022), but also highlight the potentially substantial and adverse impacts of air temperature on mental health. The impact of weather on the social environment and the behaviours conducive to that environment provides a useful framework in which to consider the pathways by which weather and climate may impact mental health.

A similar framework may be adopted when examining the influence of precipitation on mental health. Only 11 participants perceived there to be a relationship between precipitation and mental health, all of whom focused solely on rainfall, suggesting that rainfall might exacerbate symptoms of major depressive disorder, SAD, substance abuse disorder and bipolar disorder. While the literature does not support a correlation between rainfall and bipolar disorder (Carnie et al. 2011; Obrien et al. 2014; Wang et al. 2014), there is evidence to suggest that SAD and substance abuse disorder may be impacted by precipitation (Sher 2004; Xu et al. 2020). Participants suggested that weather characterised by persistent rain may lead to feelings of isolation and antisocial behaviour, encouraging individuals to remain at home, divorced from their social networks (Sher 2004). Xu et al. (2020) reported a statistical relationship between self-reported depressive symptoms and precipitation, which was argued to be due to the cloud cover causing a reduction in sunlight exposure. Sher (2004) suggests that social factors influenced by seasonal and climatic variation may encourage substance abuse. These findings provide support for the arguments made by participants in this study, who suggested that the impact of precipitation on mental health could be both direct and indirect, providing reason to consider both biological and social pathways when discussing the mechanism through which rainfall may affect mental health. Compared to other weather variables, precipitation can be considered the least significant variable, which is an argument supported by both the findings of this study, and previous research (Carnie et al. 2011; Obrien et al. 2014; Wang et al. 2014).

Research suggests that exposure to sunlight may impact schizophrenia, bipolar disorder, and major depressive disorder, as these conditions are all affected by circadian dysfunction, serotonin dysregulation, and a deficiency in vitamin D (Dominiak et al. 2015; Aguglia et al. 2017, 2019). Research suggests that light therapy benefits symptoms of bipolar depression, major depressive disorder, SAD, and schizophrenia (Benedetti et al. 2001; Dominiak et al. 2015). All 50 participants interviewed in this study were aware of the relationship between sunshine and mental health and perceived the influence of sunshine on various mental health conditions to be considerable. Utilising their professional knowledge of SAD, and the aetiologies of schizophrenia, bipolar disorder, and major depressive disorder, participants were able to recognise the important influence that sunlight exerts on mental health (Gu et al. 2019; Montes et al. 2021). Despite the significance of the influence of sunlight on mental health, some participants argued that the impacts of sunlight on mental health conditions are often supplementary, and exacerbate symptoms precipitated by either biological or socioeconomic risk factors (Traffanstedt et al. 2016). The potentially detrimental effects of sunlight on mental health as purported by some research studies (Vyssoki et al. 2012, 2014; Bauer et al. 2015; Aguglia et al. 2019), however was not discussed by participants, raising questions around the impact of such research. The findings of this study support the findings of previous research which suggests that sunlight is an important variable to consider when examining the relationship between weather, climate and mental health.

The impacts of extreme weather events were well-understood by all participants, and participants’ responses aligned with the findings of previous research, suggesting that experiencing extreme weather events adversely impacts mental health, increasing rates of PTSD, GAD, major depressive disorder, and panic disorders (Cruz et al. 2020; Barkin et al. 2021; Wu et al. 2022). Social workers, and emergency physicians spoke most on the impacts of extreme weather events on mental health and drew on their professional experiences and knowledge to inform their responses. The extreme weather event mentioned most by participants was flooding, with participants suggesting that floods affect mental health by causing psychological distress, physical harm, damage to property, and the destruction of social support structures. Participants also suggested that extreme heat events may pose a threat to individuals diagnosed with mental health conditions, due to the thermoregulatory abnormalities common in individuals with mental health conditions (Lõhmus 2018). Participants’ responses presented a holistic view of the effects of extreme weather events, highlighting the ways in which extreme weather events harm individuals physically, psychologically, and socially, with a focus on individuals’ psychosocial response to the damage and trauma caused by extreme weather events (Zhong et al. 2018; Barkin et al. 2021; Wu et al. 2022). Participants displayed a strong understanding of the pathways by which extreme weather events cause psychological distress and emphasised the importance of providing victims with both financial and social support in the aftermath of an extreme weather events. Echoing the findings of research by Simpson et al. (2011), participants suggested that even when no physical injuries are sustained following an extreme weather event, individuals can still develop PTSD, GAD, and major depressive disorder. While healthcare practitioners were not formally taught about the impact of air temperature and precipitation on mental health, it was clear that many of the participants were well-informed regarding the impact of extreme weather events on mental health, and that they had experience treating patients who had experienced an extreme weather events.

The findings of this study should be examined with the following limitations in mind. Healthcare practitioners’ perceptions may change with time, as they are influenced by the participants’ professional experiences. Short-term perceptions do not aid in developing conclusive, long-term assessments of the potential relationship between weather, climate and mental health, and are thus not conducive for developing long-term patterns for modelling purposes. The impact of weather and climate on mental health can depend, in part, on the climate of the study site, which may limit these findings to this study area, making it difficult to suggest the nature of this relationship elsewhere. To test the importance of this factor, future research could expand the scope of this study, engaging mental health practitioners across a larger range of climatic zones. It should be noted, however, that South Africa does have a considerable climatic range (Fig. 1; Karmalkar et al. 2012). While the total number of participants represent a small proportion of all healthcare practitioners in each category, there was considerable repetition in themes throughout participants’ responses suggesting that sample saturation had been reached. Selection bias may have been introduced into the study through the use of convenience sampling to identify initial participants, which potentially impacts the degree to which the sample group is representative of the target population. Thematic analysis was employed to analyse the data, and it should be noted that this method of data analysis is heavily reliant on the researcher’s interpretation of the themes within the dataset. Reflexivity was employed to ensure that the interpretation of the data was as objective as possible, and that no biases were projected onto the themes.

## Conclusion

This study aimed to explore whether mental health practitioners perceive there to be a link between mental health and daily, seasonal, or inter-annual shifts in various meteorological and climatic variables in South Africa, and the timing and causal mechanisms thereof. All 50 participants perceived there to be a link between weather and climate and specific mental health conditions, but participants perceptions of the significance of this link varied. The mental health conditions that participants perceived weather and climate to potentially influence included SAD, major depressive disorder, GAD, PTSD, schizophrenia, bipolar disorder, and substance abuse disorder. The weather and climatic variables which the majority of participants perceived to affect mental health included sunlight, air temperature, precipitation, and extreme weather events. The findings of this study suggest that the potential relationship between weather, climate and mental health has not been incorporated into the formal medical education syllabus, beyond the provisions of SAD. Participants did not consider the potential effects of weather and climate on mental health when treating and managing patients, and one could hypothesise that this is because participants were not formally educated about the potential effects of weather and climate on mental health, and thus rely on treatments that they were formally educated on, and thus either trust more or feel more secure in utilising. More research is needed on the relationship between weather, climate and mental health within a South African context.

## Data Availability

Due to ethics requirements, the data are not directly available, but can be accessed on request, provided that such request is approved by the ethics board.

## References

[CR1] Adeoye-Olatunde OA, Olenik NL (2021) Research and scholarly methods: Semi‐structured interviews. J Am Coll Clin Pharm 4:1358–1367. 10.1002/jac5.1441

[CR2] Aguglia A, Borsotti A, Cuniberti F et al (2017) The influence of sunlight exposure on hospitalization in emergency psychiatry. Chronobiol Int 34:1413–1422. 10.1080/07420528.2017.1374286 29144157 10.1080/07420528.2017.1374286

[CR3] Aguglia A, Serafini G, Escelsior A et al (2019) Maximum temperature and Solar Radiation as predictors of Bipolar patient admission in an Emergency Psychiatric Ward. Int J Env Res Pub He 16:1140. 10.3390/ijerph16071140 10.3390/ijerph16071140 PMC6480941 30934957

[CR4] Allen J, Balfour R, Bell R, Marmot M (2014) Social determinants of mental health. Int Rev Psychiatr 26:392–407. 10.3109/09540261.2014.928270 10.3109/09540261.2014.928270 25137105

[CR5] Atwoli L, Muhia J, Merali Z (2022) Mental health and climate change in Africa. BJPsych Int 19:86–89. 10.1192/bji.2022.14

[CR6] Barkin JL, Buoli M, Curry CL et al (2021) Effects of extreme weather events on child mood and behavior. Develop Med Child Neuro 63:785–790. 10.1111/dmcn.14856 10.1111/dmcn.14856 PMC8252647 33720406

[CR7] Basu R, Gavin L, Pearson D et al (2018) Examining the Association between Apparent temperature and Mental Health-Related Emergency Room visits in California. Am J Epidemiol 187:726–735. 10.1093/aje/kwx295 29020264 10.1093/aje/kwx295

[CR8] Bauer M, Glenn T, Alda M et al (2015) Influence of light exposure during early life on the age of onset of bipolar disorder. J Psychiatr Res 64:1–8. 10.1016/j.jpsychires.2015.03.013 25862378 10.1016/j.jpsychires.2015.03.013

[CR9] Benedetti F, Colombo C, Barbini B et al (2001) Morning sunlight reduces length of hospitalization in bipolar depression. J Affect Disorders 62:221–223. 10.1016/S0165-0327(00)00149-X 11223110 10.1016/s0165-0327(00)00149-x

[CR10] Brandl EJ, Lett TA, Bakanidze G et al (2018) Weather conditions influence the number of psychiatric emergency room patients. Int J Biometeorol 62:843–850. 10.1007/s00484-017-1485-z 29204686 10.1007/s00484-017-1485-z

[CR11] Braun V, Clarke V (2006) Using thematic analysis in psychology. Qual Res Psychol 3:77–101. 10.1191/1478088706qp063oa

[CR12] Bulbena A, Pailhez G, Aceña R et al (2005) Panic anxiety, under the weather? Int J Biometeorol 49:238–243. 10.1007/s00484-004-0236-0 15726446 10.1007/s00484-004-0236-0

[CR13] Bundo M, De Schrijver E, Federspiel A et al (2021) Ambient temperature and mental health hospitalizations in Bern, Switzerland: a 45-year time-series study. PLoS ONE 16:e0258302. 10.1371/journal.pone.0258302 34637463 10.1371/journal.pone.0258302 PMC8509878

[CR14] Byansi W, Galvin M, Chiwaye L et al (2023) Adverse childhood experiences, traumatic events, and mental health among adults at two outpatient psychiatric facilities in Johannesburg, South Africa: a cross-sectional analysis. BMC Psychiatry 23:581. 10.1186/s12888-023-05085-0 37563695 10.1186/s12888-023-05085-0 PMC10413614

[CR15] Carnie T-L, Berry HL, Blinkhorn SA, Hart CR (2011) In their own words: young people’s mental health in drought-affected rural and remote NSW: YOUNG PEOPLE’s MENTAL HEALTH IN DROUGHT. Aust J Rural Health 19:244–248. 10.1111/j.1440-1584.2011.01224.x 21933366 10.1111/j.1440-1584.2011.01224.x

[CR16] Chersich MF, Wright CY (2019) Climate change adaptation in South Africa: a case study on the role of the health sector. Global Health 15:22. 10.1186/s12992-019-0466-x 30890178 10.1186/s12992-019-0466-x PMC6423888

[CR17] Clayton S (2021) Climate change and mental health. Curr Envir Health Rpt 8:1–6. 10.1007/s40572-020-00303-3 10.1007/s40572-020-00303-3 33389625

[CR18] Compton MT, Shim RS (2015) The Social Determinants of Mental Health. FOC 13:419–425. 10.1176/appi.focus.20150017

[CR19] Craig A, Rochat T, Naicker SN et al (2022) The prevalence of probable depression and probable anxiety, and associations with adverse childhood experiences and socio-demographics: a national survey in South Africa. Front Public Health 10:986531. 10.3389/fpubh.2022.986531 36388391 10.3389/fpubh.2022.986531 PMC9650309

[CR20] Cruz J, White PCL, Bell A, Coventry PA (2020) Effect of Extreme Weather events on Mental Health: a narrative synthesis and Meta-analysis for the UK. Int J Public Health 17:8581. 10.3390/ijerph17228581 10.3390/ijerph17228581 PMC7699288 33227944

[CR21] Docrat S, Besada D, Cleary S et al (2019) Mental health system costs, resources and constraints in South Africa: a national survey. Health Policy Plann 34:706–719. 10.1093/heapol/czz085 10.1093/heapol/czz085 PMC6880339 31544948

[CR22] Dominiak M, Swiecicki L, Rybakowski J (2015) Psychiatric hospitalizations for affective disorders in Warsaw, Poland: Effect of season and intensity of sunlight. Psychiat Res 229:287–294. 10.1016/j.psychres.2015.07.011 10.1016/j.psychres.2015.07.011 26189339

[CR23] Etikan I, Bala K (2017) Sampling and sampling methods. Biom Biostat Int J.5:10.15406/bbij.2017.05.00149.

[CR24] Fitchett JM, Robinson D, Hoogendoorn G (2016) Climate suitability for tourism in South Africa. J Sustain Tour 25:851–867. 10.1080/09669582.2016.1251933

[CR25] Flisher AJ, Dawes A, Kafaar Z et al (2012) Child and adolescent mental health in South Africa. J Child Adol Ment Health 24:149–161. 10.2989/17280583.2012.735505 10.2989/17280583.2012.735505 25860182

[CR26] Gu S, Huang R, Yang J et al (2019) Exposure-lag-response association between sunlight and schizophrenia in Ningbo, China. Environ Pollut 247:285–292. 10.1016/j.envpol.2018.12.023 30685669 10.1016/j.envpol.2018.12.023

[CR27] Jahan S, Wraith D, Dunne MP et al (2020) Seasonality and schizophrenia: a comprehensive overview of the seasonal pattern of hospital admissions and potential drivers. Int J Biometeorol 64:1423–1432. 10.1007/s00484-020-01910-3 32281005 10.1007/s00484-020-01910-3

[CR28] Jahan S, Wraith D, Dunne MP et al (2021) Assessing evidence for seasonality of hospital admissions for schizophrenia in Queensland, Australia: a time series observational study. Int J Biometeorol 65:2025–2035. 10.1007/s00484-021-02160-7 34110485 10.1007/s00484-021-02160-7

[CR29] Karmalkar A, McSweeney C, New A et al (2012) UNDP climate change country profiles: South Africa. UNDP. https://www.geog.ox.ac.uk/research/climate/projects/undp-cp/?country=South_Africa&d1=Reports. Accessed 19 March 2023

[CR30] Lee S, Lee H, Myung W et al (2018) Mental disease-related emergency admissions attributable to hot temperatures. Sci Total Environ 616–617:688–694. 10.1016/j.scitotenv.2017.10.260 10.1016/j.scitotenv.2017.10.260 29126638

[CR31] Liu J, Varghese BM, Hansen A et al (2021) Is there an association between hot weather and poor mental health outcomes? A systematic review and meta-analysis. Environ Int 153:106533. 10.1016/j.envint.2021.106533 33799230 10.1016/j.envint.2021.106533

[CR32] Lõhmus M (2018) Possible Biological Mechanisms Linking Mental Health and Heat—A contemplative review. Int J Env Res Pub He 15:1515. 10.3390/ijerph15071515 10.3390/ijerph15071515 PMC6068666 30021956

[CR33] Maes M, De Meyer F, Peeters D et al (1993) The periodicities in and biometeorological relationships with bed occupancy of an acute psychiatric ward in Antwerp, Belgium. Int J Biometeorol 37:78–82. 10.1007/BF01214385 8330944 10.1007/BF01214385

[CR34] Magnusson A, Boivin D (2003) Seasonal affective disorder: an overview. Chronobiol Int 20:189–207. 10.1081/CBI-120019310 12723880 10.1081/cbi-120019310

[CR35] McWilliams S, Kinsella A, O’Callaghan E (2014) Daily weather variables and affective disorder admissions to psychiatric hospitals. Int J Biometeorol 58:2045–2057. 10.1007/s00484-014-0805-9 24599495 10.1007/s00484-014-0805-9

[CR36] Melrose S (2015) Seasonal Affective Disorder: An Overview of Assessment and Treatment Approaches. Depress Res Treat 2015:1–6. 10.1155/2015/178564 10.1155/2015/178564 PMC4673349 26688752

[CR37] Montes JM, Serrano C, Pascual-Sanchez A (2021) The influence of weather on the course of bipolar disorder: a systematic review. Eur J Psychiatry 35:261–273. 10.1016/j.ejpsy.2021.03.002

[CR38] Mullins JT, White C (2019) Temperature and mental health: evidence from the spectrum of mental health outcomes. J Health Econ 68:102240. 10.1016/j.jhealeco.2019.102240 31590065 10.1016/j.jhealeco.2019.102240

[CR39] Muneer A (2016) The neurobiology of bipolar disorder: an Integrated Approach. Chonnam Med J 52:18. 10.4068/cmj.2016.52.1.18 26865997 10.4068/cmj.2016.52.1.18 PMC4742607

[CR40] Muntaner C, Ng E, Prins SJ et al (2015) Social Class and Mental Health: Testing Exploitation as a relational determinant of Depression. Int J Health Serv 45:265–284. 10.1177/0020731414568508 25813501 10.1177/0020731414568508 PMC4747250

[CR41] Nguse S, Wassenaar D (2021) Mental health and COVID-19 in South Africa. S Afr J Psychol 51:304–313. 10.1177/00812463211001543 38603189 10.1177/00812463211001543 PMC8107260

[CR42] Nicholson S (2000) The nature of rainfall variability over Africa on time scales of decades to millenia. Global Planet Change 26:137–158. 10.1016/S0921-8181(00)00040-0

[CR43] Niu Y, Gao Y, Yang J et al (2020) Short-term effect of apparent temperature on daily emergency visits for mental and behavioral disorders in Beijing, China: a time-series study. Sci Total Environ 733:139040. 10.1016/j.scitotenv.2020.139040 32446053 10.1016/j.scitotenv.2020.139040 PMC7298617

[CR44] OBrien LV, Berry HL, Coleman C, Hanigan IC (2014) Drought as a mental health exposure. Environ Res 131:181–187. 10.1016/j.envres.2014.03.014 24727641 10.1016/j.envres.2014.03.014

[CR45] Parker GB, Brotchie H, Graham RK (2017) Vitamin D and depression. J Affect Disorders 208:56–61. 10.1016/j.jad.2016.08.082 27750060 10.1016/j.jad.2016.08.082

[CR46] Pillay Y (2019) State of mental health and illness in South Africa. S Afr J Psychol 49:463–466. 10.1177/0081246319857527

[CR47] Posel D, Oyenubi A, Kollamparambil U (2021) Job loss and mental health during the COVID-19 lockdown: evidence from South Africa. PLoS ONE 16:e0249352. 10.1371/journal.pone.0249352 33784339 10.1371/journal.pone.0249352 PMC8009396

[CR48] Potgieter A, Fabris-Rotelli IN, Breetzke G, Wright CY (2022) The association between weather and crime in a township setting in South Africa. Int J Biometeorol 66:865–874. 10.1007/s00484-022-02242-0 35061073 10.1007/s00484-022-02242-0

[CR49] Robertson LJ, Bouwer JC (2024) Mental health services in Gauteng, South Africa: a proxy evaluation using pharmaceutical data. S Afr J Psychiatr 30:2157–2166. 10.4102/sajpsychiatry.v30i0.2157 38628901 10.4102/sajpsychiatry.v30i0.2157 PMC11019062

[CR50] Roffe SJ, Fitchett JM, Curtis CJ (2021) Quantifying rainfall seasonality across South Africa on the basis of the relationship between rainfall and temperature. Clim Dyn 56:2431–2450. 10.1007/s00382-020-05597-5

[CR51] Schneider A, Hampel R, Ladwig K-H et al (2020) Impact of meteorological parameters on suicide mortality rates: a case-crossover analysis in Southern Germany (1990–2006). Sci Total Environ 707:136053. 10.1016/j.scitotenv.2019.136053 31863976 10.1016/j.scitotenv.2019.136053

[CR52] Schutte FH, Breetzke GD (2018) The influence of extreme weather conditions on the magnitude and spatial distribution of crime in tshwane (2001–2006). S Afr Geogr J 100:364–377. 10.1080/03736245.2018.1498384

[CR53] Sher L (2004) Alcoholism and seasonal affective disorder. Compr Psychiatry 45:51–56. 10.1016/j.comppsych.2003.09.007 14671737 10.1016/j.comppsych.2003.09.007

[CR54] Simpson DM, Weissbecker I, Sephton SE (2011) Extreme weather-related events: implications for mental health and well-being. International Culture and Psychology. p. 57–78.

[CR55] Sung T-I, Chen M-J, Lin C-Y et al (2011) Relationship between mean daily ambient temperature range and hospital admissions for schizophrenia: results from a national cohort of psychiatric inpatients. Sci Total Environ 410–411:41–46. 10.1016/j.scitotenv.2011.09.028 10.1016/j.scitotenv.2011.09.028 22018962

[CR57] Tim N, Zorita E, Hünicke B et al (2022) The impact of the Agulhas current system on precipitation in southern Africa in regional climate simulations covering the recent past and future. Weather Clim Dynamics Discuss 4:381–397. 10.5194/wcd-4-381-2023

[CR56] Traffanstedt MK, Mehta S, LoBello SG (2016) Major Depression with Seasonal Variation: is it a valid construct? Clin Psychol Sci 4:825–834. 10.1177/2167702615615867

[CR59] Vyssoki B, Praschak-Rieder N, Sonneck G et al (2012) Effects of sunshine on suicide rates. Compr Psychiat 53:535–539. 10.1016/j.comppsych.2011.06.003 21821241 10.1016/j.comppsych.2011.06.003

[CR58] Vyssoki B, Kapusta ND, Praschak-Rieder N et al (2014) Direct effect of Sunshine on suicide. Jama Psychiat 71:1231. 10.1001/jamapsychiatry.2014.1198 10.1001/jamapsychiatry.2014.1198 25208208

[CR61] Wang X, Lavigne E, Ouellette-kuntz H, Chen BE (2014) Acute impacts of extreme temperature exposure on emergency room admissions related to mental and behavior disorders in Toronto, Canada. J Affect Disorders 155:154–161. 10.1016/j.jad.2013.10.042 24332428 10.1016/j.jad.2013.10.042

[CR60] Wang S, Zhang X, Xie M et al (2018) Effect of increasing temperature on daily hospital admissions for schizophrenia in Hefei, China: a time-series analysis. Pub Health 159:70–77. 10.1016/j.puhe.2018.01.032 29567010 10.1016/j.puhe.2018.01.032

[CR62] Wu Y, Yao Z, Ma G et al (2022) Effects of extreme precipitation on hospitalization risk and disease burden of schizophrenia in urban and rural Lu’an, China, from 2010 to 2019. Environ Sci Pollut Res 29:19176–19184. 10.1007/s11356-021-16913-9 10.1007/s11356-021-16913-9 34713403

[CR63] Xu C, Wu W, Peng-Li D et al (2020) Intraday weather conditions can influence self-report of depressive symptoms. J Psychiat Res 123:194–200. 10.1016/j.jpsychires.2020.02.006 32086180 10.1016/j.jpsychires.2020.02.006

[CR64] Yi W, Zhang X, Gao J et al (2019) Examining the association between apparent temperature and admissions for schizophrenia in Hefei, China, 2005–2014: a time-series analysis. Sci Total Environ 672:1–6. 10.1016/j.scitotenv.2019.03.436 30954808 10.1016/j.scitotenv.2019.03.436

[CR65] Zhang S, Yang Y, Xie X et al (2020) The effect of temperature on cause-specific mental disorders in three subtropical cities: a case-crossover study in China. Environ Int 143:105938. 10.1016/j.envint.2020.105938 32688157 10.1016/j.envint.2020.105938

[CR66] Zheng G, Li K, Wang Y (2019) The effects of High-Temperature Weather on Human Sleep Quality and Appetite. Int J Env Res Pub He 16:270. 10.3390/ijerph16020270 10.3390/ijerph16020270 PMC6351950 30669302

[CR67] Zhong S, Yang L, Toloo S et al (2018) The long-term physical and psychological health impacts of flooding: a systematic mapping. Sci Total Environ 626:165–194. 10.1016/j.scitotenv.2018.01.041 29339262 10.1016/j.scitotenv.2018.01.041

